# Monte Carlo Simulation of Double-Strand Break Induction and Conversion after Ultrasoft X-rays Irradiation

**DOI:** 10.3390/ijms222111713

**Published:** 2021-10-28

**Authors:** Ya-Yun Hsiao, Fang-Hsin Chen, Chun-Chieh Chan, Ching-Chih Tsai

**Affiliations:** 1 Department of Radiology, Chung Shan Medical University Hospital, Taichung 40201, Taiwan; yhsiao@csmu.edu.tw; 2 Department of Medical Imaging and Radiological Sciences, Chung Shan Medical University, Taichung 40201, Taiwan; 3 Department of Medical Imaging and Radiological Sciences, Chang Gung University, Taoyuan 33302, Taiwan; fanghsinchen@mail.cgu.edu.tw; 4 Radiation Biology Research Center, Institute for Radiological Research, Chang Gung University, Taoyuan 33302, Taiwan; 5 Department of Radiation Oncology, Chang Gung Memorial Hospital-Linkou Branch, Taoyuan 33305, Taiwan; 6 Department of Electrical Engineering, National Chung Hsing University, Taichung 40227, Taiwan

**Keywords:** DSB induction, ultrasoft X-rays, DNA repair, enzymatic DSB

## Abstract

This paper estimates the yields of DNA double-strand breaks (DSBs) induced by ultrasoft X-rays and uses the DSB yields and the repair outcomes to evaluate the relative biological effectiveness (*RBE*) of ultrasoft X-rays. We simulated the yields of DSB induction and predicted them in the presence and absence of oxygen, using a Monte Carlo damage simulation (MCDS) software, to calculate the *RBE*. Monte Carlo excision repair (MCER) simulations were also performed to calculate the repair outcomes (correct repairs, mutations, and DSB conversions). Compared to ^60^Co γ-rays, the *RBE* values for ultrasoft X-rays (titanium K-shell, aluminum K-shell, copper L-shell, and carbon K-shell) for DSB induction were respectively 1.3, 1.9, 2.3, and 2.6 under aerobic conditions and 1.3, 2.1, 2.5, and 2.9 under a hypoxic condition (2% O_2_). The *RBE* values for enzymatic DSBs were 1.6, 2.1, 2.3, and 2.4, respectively, indicating that the enzymatic DSB yields are comparable to the yields of DSB induction. The synergistic effects of DSB induction and enzymatic DSB formation further facilitate cell killing and the advantage in cancer treatment.

## 1. Introduction

The relative biological effectiveness (*RBE*) depends on photon energy. In particular, *RBE* of ultrasoft X-rays (<20 keV) increases with decreasing energy [1,2,3]. Ultrasoft X-rays are more effective up to a factor of 4 than 250 keV X-rays with regard to double-strand break (DSB) yield, cell death, and mutations [4,5,6]. The higher *RBE* is on account of the short traveling distances (~7 nm) of the secondary electrons [7], which is similar to the width of DNA helix. These very-low-energy electrons which produce through photoelectric interactions or secondary electrons scattering [8] cause highly clustered energy depositions and are the predominant contributor to complex DNA damage, which leads to an increment in genetic mutation, DNA damage yield, and apoptosis.

DSBs can be directly induced by energy deposition or from the outcome of a mis-repairing process dealing with non-DSB clustered lesions via base excision repair (BER) pathways [9,10] and nucleotide excision repair (NER) pathways [11,12,13], known as enzymatic DSBs. Monte Carlo simulations and experiments performed on cell lines demonstrate that the yield of enzymatic DSB is comparable to the yield of direct DSB induction, which is also known as prompt DSB yield [14,15]. These extra DSBs that are formed after the mis-repair process contribute further to the DSB yield and result in a higher *RBE* value [16]. In these cases, the greater numbers of DSBs and damage clusters increase the DNA damage complexity [17].

Most clustered damage on DNA involves the presence of O_2_ [17]. When clustered DNA damage occurs under aerobic conditions, it is firstly induced by free radicals and then modified by dissolved O_2._ Thus, if O_2_ is present when the DNA damage occurs, the damage is fixed and cannot be reversible repaired by chemical repair by thiols, which is widely regarded as the oxygen fixation hypothesis [18]. However, the O_2_ does not fix the damage directly; rather, it modifies the pathway and final chemical products [19].

In general, ultrasoft X-rays are not used for conventional X-ray therapy (XT) because they penetrate to a shallower depth [7]. However, they are used for dermatological radiotherapy, known as Grenz therapy (GT) [20]. GT is used to reduce allergic skin conditions such as eczema, contact dermatitis and psoriasis, etc. [20,21]. It can also be used to treat malignant cells such as lentigo maligna (LM) and LM melanoma [22]. In contrast to XT, GT has very few side effects, and its most adverse effects are erythema and pigmentation [23]. Since 1959, however, GT has been reported to induce carcinoma, and the pattern of tumor development is similar to that resulting from exposure to ultraviolet light [23].

For cells that are irradiated by GT, the processes of DSB yield are almost exclusively due to the low-energy secondary electrons [24], and the data obtained from simulations of GT or low-energy secondary electrons [25,26] show that these electrons could produce higher yields of complex DSB damage than those induced by XT, leading to other varieties of DSB [27,28]. Here, we present a study covering DSB induction, DNA repair, and the effects of O_2_ on damage yields for cells irradiated by GT and further investigate the *RBE* of DSB induction and DNA repair for cells irradiated by GT.

## 2. Materials and Methods

### 2.1. Irradiation Geometries and Materials

A cylindrical geometry has been used to mimic the typical cell culture experiments [29,30,31]. The cylinder was designed with a radius of 26 mm and a height of 10 μm (the thickness of a mammalian cell) to mimic a monolayer cell culture geometry. The photon sources were put 5 μm above the cells and were set like a pencil beam incident on the cylinder. The cell was approximated by water at a density of 1.0 g cm^−3^, as used in a previous study [29].

### 2.2. Determination of Secondary-Electron Fluence

The PENCYL program in the version 2011 penetration and energy loss of positrons and electron (PENELOPE) Monte Carlo code [32,33,34] was used to calculate the fluence of secondary electrons passing over single cells. All simulations were performed with at least 2 × 10^8^ source particle histories, using the 2011 version of PENELOPE. All primary and secondary electrons were followed down to 50 eV [35]. PENELOPE uses a scattering model [32] that merges analytical cross-sectional models for different interaction mechanisms with numerical databases. The modified version of PENELOPE 2011 uses a more realistic continuous energy-loss distribution to replace the previous discrete optical oscillator-strength model, which increases both the reliability and the generality of the code.

The simulation of electron transport processes in PENELOPE was controlled by the parameters C_1_, C_2_, W_CC_, and W_CR_ whose definitions and roles were explained previously [36]. Briefly, C_1_ controls the average angular deflection that is produced by multiple elastic scattering of electrons along the step between hard events and C_2_ controls the maximum average fractional energy loss in the step. W_CC_ and W_CR_ respectively represent the cut-off energy loss for hard inelastic collisions and for hard Bremsstrahlung emission. The values of parameters in this study were set as C_1_ = 0.00, C_2_ = 0.01, W_CC_ = 10 eV, and W_CR_ = 10 eV, as suggested for the simulation of very low energy (0.1–10 keV) electrons in microdosimetry applications [37].

### 2.3. Monte Carlo Damage Simulation (MCDS)

The MCDS is a fast algorithm that calculates the yield of clustered damage within a cell that is irradiated with photons, mono-energetic electrons, protons, or heavy ions, such as ^56^Fe [27,38,39]. The MCDS software is free and available online for commercial, educational, or research purposes [39] and simulates the induction and clustering of DNA lesions in both hypoxic and normoxic cells (>21% O_2_) [27,38,39].

The MCDS is not a track structure simulation code and its algorithm generates DNA damage data similar to those using computationally-expensive but detailed event-by-event simulations. The types of DNA damage can be grouped into base damage (BD), single strand breaks (SSBs), and DSB. Various complex SSB or DSB, i.e., SSB^+^, 2SSB, DSB^+^ and DSB^++^, and total SSB and DSB were defined previously [40]. Briefly, the types and classifications of DNA damage and their abbreviations are: two or more strand breaks on the same strand (SSB^+^), two or more strand breaks on opposite strands that do not constitute DSB (2SSB), DSBs with additional break(s) on a strand within 10 base pairs (DSB^+^), and more than one DSB within 10 base pairs (DSB^++^).

### 2.4. Monte Carlo Excision Repair (MCER) Simulation

The MCER simulation was used to estimate the probabilities of the repair outcomes in the BER and BER/NER pathways for DNA damage in cells that are irradiated with electrons, protons, and helium ions [41,42] and has been used for several studies [43,44]. The MCER simulation simulated the key steps of repair pathways, i.e., short-patch BER (SP BER), long-patch BER (LP BER), SP BER/NER, and LP BER/NER [41,42]. A single nucleotide replacement generally occurs in the SP BER pathway and the LP BER pathway removes a fragment of 2–13 nucleotides. MCER output the probabilities of the repair outcomes that a DNA clustered damage repair correctly, repair with a mutation and converted into a DSB.

The following input parameters were used and are the same as those for a previous study [42]: inhibition distance = 8 base pairs, probability of selecting a lesion from the first strand break = 0.5, polymerase error for SP BER = 1.0^−4^, polymerase error for LP BER and for NER = 1.0^−6^, probability of incorrect insertion opposite a damaged base = 0.75, and probability of incorrect insertion opposite a lost base = 0.75. All four pathways (SP BER, LP BER, NER/SP BER, and NER/LP BER) were simulated. The results show that the NER/LP BER pathway has the lowest probability of correct repair and highest probability for mutation and DSB formation, which is similar to the results for ^60^Co γ-rays [44], protons [40], and helium ions [44]. These results are ascribed to the fact that the repair of non-DSB clusters occurs mainly through the BER pathway, but other bulk damage is mended by the NER pathway. Therefore, the LP BER pathway was selected to represent enzymatic DSB.

### 2.5. Calculation of DSB Conversion from DNA Damage

The enzymatic DSBs for ultrasoft X-rays and ^60^Co γ-rays were calculated with the dose-weighted formula [29]:

(1) $$Y_{i}=\frac{\int_{0}^{\infty }dEY_{i}(E)p_{i}(E)\Phi (E){LET}_{\infty }(E)}{\int_{0}^{\infty }dE\Phi (E){LET}_{\infty }\left(E\right)}$$

where Φ(*E*) is the energy fluence of secondary electrons of energy *E* that are generated in the cell medium from ultrasoft X-rays or ^60^Co photon interactions. The value for unrestricted *LET* (stopping power) from the National Institute of Standards and Technology is used for electrons with energy that is greater than 1 keV [45]. For energies below 1 keV, the *LET* was calculated by fitting to the data of Emfietzoglou and Nikjoo (2007) [46], i.e.,

(2) $$LET(\mathrm{eV}/\mathrm{nm})=4376T^{-1}\ln T-19803T^{-1}+129622T^{-2}$$

where *T* is the electron energy in eV.

### 2.6. Hypoxia Reduction Factor (HRF) and RBE

The oxygen enhancement ratio is generally used to quantify the effects of oxygen concentration on DNA damage and cell killing and defined as the ratio of the hypoxic dose to the aerated dose that is required to achieve the same biological outcome [18]. Some studies also used it as a scaling factor to quantify the biological outcomes or radiation sensitivity of cells under hypoxia conditions [47,48]. To avoid inconsistency, we used another term, *HRF*, previously defined as the ratio of DSB yield under aerobic conditions (21% O_2_) to that under hypoxic conditions [47] to quantify the effects of oxygen concentration on DNA damage induction, i.e.,

(3) $$HRF=\frac{\Sigma_{a}}{\Sigma_{h}}$$

where the DSB yield Σ with the subscript *a* and *h*, respectively denote aerobic and hypoxia conditions.

The *RBE* is originally defined as the ratio of the dose of low *LET* reference radiation to the dose of any other radiation that is required to achieve an equal biological effect [18]. The *RBE* can also be interpreted as the ratio of the DSB yields of cells irradiated with different radiation sources because DSB induction is linearly proportional to the absorbed dose, *D,* up to a hundred Gy under aerobic condition (21% O_2_) and severe hypoxia (0.1% O_2_) [49,50], as shown below:

(4) $$RBE=\frac{D_{\gamma }}{D_{U}}=\frac{\Sigma_{U}}{\Sigma_{\gamma }}$$

The subscripts, *U* and *γ*, respectively denote ultrasoft X-rays and γ-rays. The DSB yield for ^60^Co γ-rays is the reference for all reported *RBE* values.

## 3. Results

The *RBE* calculated from the measured DSB induction relative to ^60^Co γ-rays increased from 1.4 to 2.7 as the photon energy decreased from 4.55 keV to 280 eV [3]. The MCDS-derived *RBE* for DSB induction agreed well (within ±5.3%) with the *RBE* values using the measured DSB induction for photon energies of 280 eV to 1.49 keV (Table 1). The yield of more complicated types of DNA damage, such as DSB^+^ and DSB^++^, increased as photon energy decreased (Table 2), resulting in an increasing yield of total DSBs as photon energy decreased. In contrast, the yield for simpler types of damage, such as BD and SSBs, decreased as photon energy decreased, thus total SSBs and total damage decreased as photon energy decreased. The increase in the yields for more complicated types of DNA damage resulted in an increase in the DNA damage complexity as photon energy decreased.

The complexity of the DNA damage was significantly affected by indirect action and oxygen. The yield of DNA damage was simulated at an oxygen concentration of 2% (Table 3) and lower (0.1% O_2_, Table 4). The limits of the standard deviations for all subtypes of DNA damage (i.e., BD, SSB, SSB^+^, 2SSB, DSB, DSB^+^, and DSB^++^) listed in Table 2, Table 3 and Table 4 are within 0.2%. The resulting yields of SSBs, DSBs, DSBs^+^, and DSBs^++^ of Table 3 and Table 4 were lower than those at an oxygen concentration of 21%, regardless of the level of photon energy (Table 2). There was no clear relationship between the yield of BD and oxygen concentration but the portion of BD of all energy levels are decreasing as oxygen concentration decreases. The yield of BD from copper K-shell and carbon K-shell photons at lower oxygen concentrations (≤2%) (Table 3 and Table 4) was slightly higher than that at an oxygen concentration of 21% (Table 2). As the oxygen concentration decreased to 2% or 0.1%, the *RBE* values estimated based on DSB induction relative to ^60^Co γ-rays were increasing to the range 1.3–2.9 and 1.4–4.4, respectively (Table 3 and Table 4).

**Figure 1:**
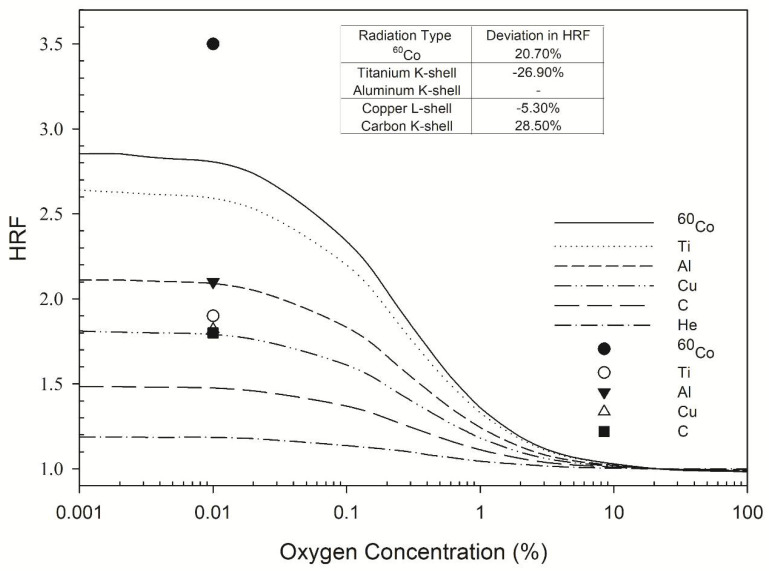
HRF for DSB induction as a function of oxygen concentration. The symbols Ti, Al, Cu, and C represent titanium K-shell, aluminum K-shell, copper L-shell, and carbon K-shell radiation, respectively. The lines indicate the Monte Carlo damage simulation results (this work) and the individual symbols represent the experimental results of de Lara et al. (2001) [3].

The *RBE* for DSB induction for cells irradiated with copper L-shell photons (0.96 keV) increased from 2.3 (Table 1) to 2.5 (8.6% increase; Table 3) and the *RBE* for cells irradiated with carbon K-shell photons (280 eV) increased from 2.7 to 2.9 (7% increase) as oxygen concentration decreases from 21% to 2%. Under severe hypoxia (0.1% O_2_), the *RBE* values for cells irradiated with copper L-shell and carbon K-shell photons further increase to 3.3 and 4.4, respectively. The *RBE* values for lower energy photons such as carbon K-shell photons (280 eV) under hypoxic conditions were greater, because there was a greater decrease in the yield of DSBs for ^60^Co γ-rays. The change in DSB yield for different oxygen concentrations is quantified using HRF, considering that carbon K-shell photons (280 eV) had a lower HRF than ^60^Co γ-rays (see Figure 1).

The highest HRF values calculated by the MCDS for ^60^Co γ-rays, titanium K-shell, aluminum K-shell, copper L-shell, and carbon K-shell X-rays were 2.9, 2.6, 2.1, 1.9, and 1.4, respectively, for an oxygen concentration of less than 0.01% (Figure 1). All of these HRF values were constant within 0–0.1% O_2_ and gradually decreased as the oxygen concentration increased to 10% O_2_. The respective measured values of the HRF for ^60^Co γ-rays, titanium K-shell, aluminum K-shell, copper L-shell, and carbon K-shell were 3.5, 1.9, 2.1, 1.8, and 1.8 [3] for an oxygen concentration of less than 0.01% (Figure 1). The measured values of the HRF for ultrasoft X-rays with different energies differed from those calculated by the MCDS by up to 28.5% (Figure 1, inset table).

**Figure 2:**
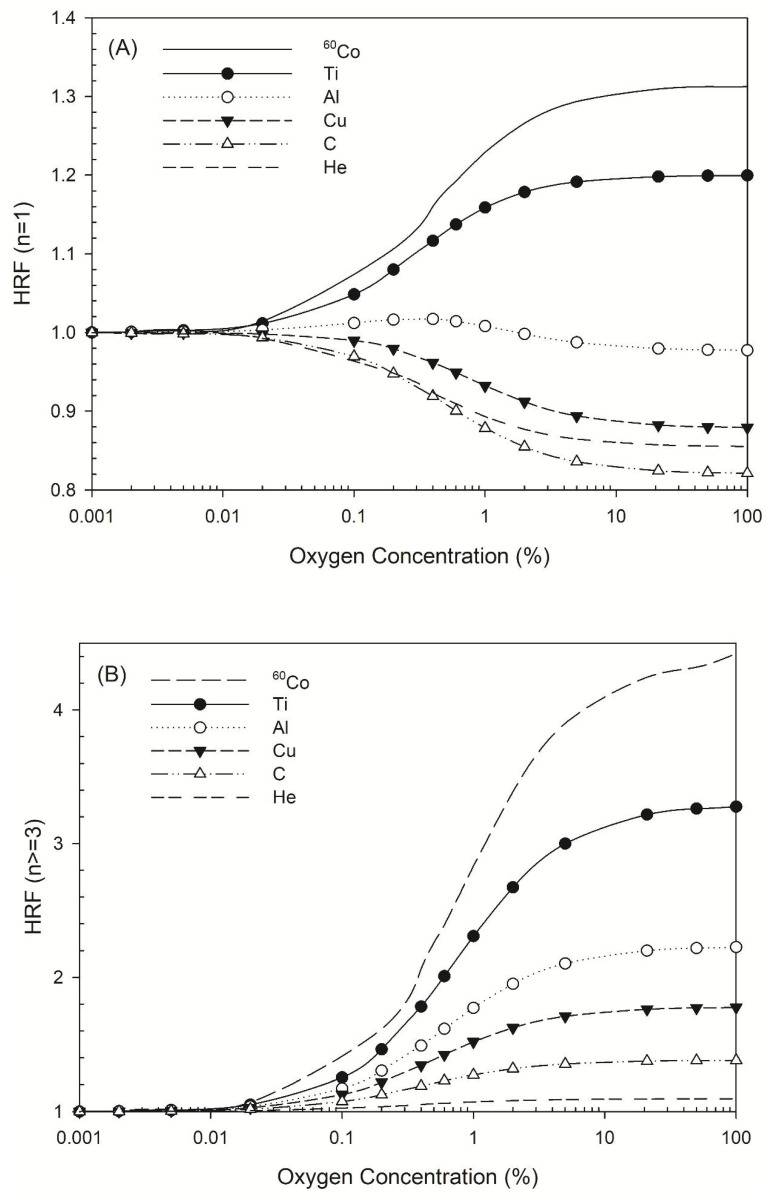
HRF for different types of radiation at various oxygen concentrations. The symbols Ti, Al, Cu, C, and He represent titanium K-shell, aluminum K-shell, copper L-shell, and carbon K-shell X-rays, and helium ions. (**A**) HRF for DNA damage with *n* = 1 lesion as a function of oxygen concentration and (**B**) HRF for DNA damage with *n* ≥ 3 lesions as a function of oxygen concentration.

The HRF for DSB induction by all radiation types considered here increased as oxygen concentration decreased, but the HRF for non-DSB clusters varied in different ways. Figure 2A shows the HRF for a non-DSB cluster with just one lesion (*n* = 1), for which the HRF for ^60^Co γ-rays and titanium K-shell X-rays increased as oxygen concentration increased, while that for aluminum K-shell, copper L-shell, carbon K-shell, and 3.31 MeV helium ions (linear energy transfer (*LET*) =120 keV/μm) [44] decreased. There was a greater decrease in the HRF for carbon K-shell X-rays than for 3.31 MeV helium ions (Figure 2A). For complex DNA damage, such as DNA with three lesions or more, the HRF for all types of radiation increased as oxygen concentration increased (Figure 2B).

In terms of DNA repair via the LP BER pathway for cells irradiated by ^60^Co γ-rays, titanium K-shell, aluminum K-shell, copper L-shell, and carbon K-shell ultrasoft X-rays, the probability of correct repair, mutation, and DSB formation were calculated (Table 5). The probability of correct repair decreased as photon energy decreased and the probability of mutation and DSB conversion increased. In regards to pathways, the combined NER/LP BER pathway had the lowest probability of correct repair and the highest probability of mutation and DSB conversion. The probability of repair outcomes for carbon K-shell X-rays was comparable to that reported for helium ions (*LET* = 120 keV/μm) [44]. The probability of correct repair, mutation, and DSB conversion for carbon K-shell X-rays was 0.822, 0.128, and 0.05, and for helium ions it was 0.78, 0.16, and 0.06, suggesting a possible application for carbon K-shell X-rays in tumor killing.

The yield for DSB conversion was less than that for DSB induction for all energy levels, because the lost proportion of DSB conversion increased as photon energy decreased (from 88% to 69% for 4.55 keV to 0.28 keV). The range of *RBE* values (1.3–2.6) for DSB induction was greater than that calculated from DSB conversion (1.6–2.4; Table 6).

## 4. Discussion

In this study, the yields of induction and conversion of DSB for cells irradiated by ultrasoft X-rays were calculated. *RBE* values were also computed under various oxygen concentrations, using ^60^Co γ-rays as the reference radiation. In the following sections, we compare our results with experimental results and discuss the effects of oxygen.

### 4.1. DNA Damage Profile

Relative to ^60^Co γ-rays, the measured *RBE* for DSB induction in cells irradiated by titanium K-shell, aluminum K-shell, copper L-shell, and carbon K-shell X-rays increased from 1.4 to 2.7 [3] as the photon energy decreased from 4.55 keV to 280 eV (Table 1), which was within a 7% difference to the simulated values. Other studies have shown that the *RBE* of MCDS-derived DSB yields were comparable (within 7.1%) to the experimentally measured yield of ultrasoft X-rays (4.55 keV to 280 eV) [29,31]. The MCDS-derived DSB yield (21.6 per Gy per Gbp) for cells irradiated with 280 eV photons in the study by Streitmatter et al. (2017) [31] gave an *RBE* of 2.7 relative to ^60^Co γ-rays (8.6 per Gy per Gbp), which was the same value as that experimentally obtained by de Lara et al. (2001) [3].

Data summarized in Table 2, Table 3 and Table 4 showed that the DSB yields for all energy levels are decreasing as oxygen concentration decreases from 21% to 0.1% O_2_. The *RBE* for DSB induction increases from 1.3–2.6 under aerobic condition (21% O_2_) to 1.3–2.9 under moderate hypoxia (2% O_2_) and further 1.4–4.4 under severe hypoxia (0.1% O_2_). These results demonstrated that oxygen plays a significant role in DSB induction via indirect action, especially for photon energy above 4.55 keV. The DSB induction for the reference radiation, ^60^Co γ-rays, reduces 53% as oxygen concentration reduces from 21% to 0.1% while that for carbon K shell (280 eV) reduces 27% only.

In addition to DSB yield, the MCDS provided specified complexity of the DNA damage, and these results were approximated to those of track structure simulations [27]. The portion of simple DNA damage, i.e., BD, declined as photon energy decreased, while that of other types of DNA damage, such as SSB^+^, 2SSB, DSBs, DSBs^+^, and DSBs^++^, increased (Table 2, Table 3 and Table 4). It has been shown that the increase in the ionization density as photon energy decreases results in an increment in the DNA damage complexity, possibly due to the increase in the *LET* of secondary low energy electrons. However, the absence of oxygen could reduce the yields of DNA damage. If oxygen is present, the DNA damage would be fixed by oxygen; otherwise thiols could repair DNA damage, such as glutathione, which has been referred as chemical repair [17]. Values in Table 2, Table 3 and Table 4 show that only BD yields of carbon K-shell photons increase as oxygen concentration decreases from 21% to 0.1% while the yields of other types of DNA damage decrease. Because higher-*LET* radiations such as carbon K-shell photons may produce more complex damage, it is possible that the chemical repair process reduces the complex DNA damage such as SSB^+^, 2SSB, or DSB to BD under hypoxia.

### 4.2. HRF Comparison

The presence of oxygen contributed to the DNA damage complexity. The yield of total DSBs for carbon K-shell X-rays (280 eV) at an oxygen concentration of 21% (Table 2) was 21.2 per Gy per Gbp, and was 20.0 per Gy per Gbp (4.5% decrease) at a 2% oxygen concentration (Table 3) and reduced to 15.5 per Gy per Gbp (27% decrease) at 0.1% oxygen concentration (Table 4). The MCDS-derived HRF values (Figure 1) were in the range of 1.4–2.6, which agreed well with the range (1.8–2.1) for V79 cells [51] for all four energy levels of the ultrasoft X-rays.

However, the HRF values obtained from a Monte Carlo simulation using track structures, which simulate the effect of oxygen, were about 3.0 for X-rays or particles with an *LET* of < 10 keV/μm [52]. Experimental data from another study also showed that the HRF value for murine squamous cell carcinoma cells irradiated with 200 kVp X-rays (*LET* ~1 keV/μm) was 1.68 [53]. The differences in HRF values between experimental data and simulations suggest that the HRF value may vary depending on cell type [52].

Our simulation data (Figure 2A) revealed the HRF values for non-DSB clustered damage with one lesion caused by ^60^Co γ-rays or titanium K-shell X-rays, aluminum K-shell, copper L-shell, and carbon K-shell X-rays. The HRF for ^60^Co γ-rays and titanium K-shell X-rays increased as oxygen concentration increased, which shows that the presence of oxygen increased the number of instances of single-lesion DNA damage due to oxygen fixation [17]. The HRF for titanium K-shell X-rays and ^60^Co γ-rays was similar, possibly due to the energy of the secondary electrons induced by titanium K-shell X-rays, which is about 4.55 keV. The DNA damage profiles are similar when the electron energy is >2 keV [54], resulting in a near-identical DSB yield for titanium K-shell X-rays and ^60^Co γ-rays [24]. In contrast, the HRF for damage that produced one lesion decreased as oxygen concentration increased for the other three types of ultrasoft X-rays, which is in accordance with helium ions, a type of high *LET* radiation (*LET* = 120 keV/μm). That may be explained that the sparse DNA damage pattern is present for low-*LET* radiations (the secondary electrons produced through the irradiation of ^60^Co γ-rays and titanium K-shell X-rays on cells), while the denser pattern is present for higher-*LET* radiations. The presence of oxygen intensifies the effects of indirect actions and it turns out that the number of DNA damage with one lesion increases as the oxygen concentration increases (Figure 2A) for low-*LET* radiations (sparse pattern). In terms of complex DNA damage, the HRF values for ultrasoft X-rays such as aluminum K-shell, copper L-shell, and carbon K-shell X-rays were 1.4–2.0, which are comparable to that for helium ions (1.1; Figure 2B). The values for titanium K-shell X-rays and ^60^Co γ-rays were 3.3 and 4.5, respectively. The HRF values decrease as photon energy decreases (Figure 2B).

### 4.3. Comparison of Repair Outcomes

The respective *RBE* values obtained from the MCER-derived probability of mutation frequency for cells that are irradiated with aluminum K-shell and carbon K-shell X-rays were 2.9 and 4.4, using ^60^Co γ-rays as the reference radiation (Table 5). The *RBE* values obtained from the measured mutation frequencies of HF19 human diploid fibroblast cells irradiated with aluminum K-shell and carbon K-shell X-rays were 2.6 and 2.2, respectively, and the *RBE* values for V79 cells were 2.1 and 3.0, respectively, using 250 keV X-rays as the reference radiation [4,5]. Accordingly, the *RBE* values for the mutation frequency of cells irradiated with aluminum K-shell X-rays were in the range of 2.1–2.6 [4,5], while that generated by the MCER simulation was 2.9. For carbon K-shell X-rays, the *RBE* values were 2.2–3.0 [4,5] and the MCER-derived *RBE* was 4.4. The disagreement between the MCER-derived results and the experimental data could be due to several factors. Cell type, cell cycle length, and assay type all affect the measured *RBE* values, which are determined using genetic effects such as mutations and aberrations [5,55]. The *RBE* for the mutation frequency of Chinese hamster V79 cells (6.1 ± 1.0 μm cell thickness) [56] irradiated with carbon K shell X-rays, relative to ^60^Co γ-rays, was 3.0 [5], but it has also been measured as 2.0–2.6 [7] for mouse C3H10T1/2 cells (2.9 ± 0.6 μm cell thickness) [56]. If chromosomal aberrations are taken into account, the *RBE* values for Chinese hamster V79 cells, baby hamster kidney (BHK) fibroblasts, and human lymphocyte cells irradiated with carbon K-shell X-rays are 2.4, 2.6, and 2.1, respectively, relative to ^60^Co γ-rays [5]. The variation in *RBE* for genetic effects could be partly attributed to the highly localized energy deposition of secondary electrons on a micrometer or nanometer scale [57,58]. Intracellular factors such as cell thickness and type also play a role [59,60]. The MCER-derived results provide useful information about repair outcomes, but may be overestimated (Table 5).

Table 6 summarizes the *RBE* values for DSB induction and enzymatic DSB yields. The *RBE* values for DSB induction and enzymatic DSBs were in the range 1.3–2.6 and 1.6–2.4, respectively, suggesting that the enzymatic DSB yields are comparable to the yields of DSB induction. Under hypoxia conditions, the enzymatic DSB yields may be reduced due to the reduction of non-DSB DNA damage (Table 2, Table 3 and Table 4), but the *RBE* values for enzymatic DSB yields may also be higher, as the *RBE* values for DSB induction, due to a higher reduction in the yields of ^60^Co γ-rays. It has been reported that the oxygen level is around 4–7.5% in most normal tissues, but reduces to 0.3–4.2% in tumors [61]. That suggests that GT has a higher *RBE* than XT under hypoxia conditions and has an advantage in cancer treatment.

## 5. Conclusions

The MCDS- and MCER-derived *RBE* values for ultrasoft X-rays estimated based on DSB induction and enzymatic DSBs under an aerobic condition are in the range of 1.3–2.6 and 1.6–2.4, respectively. The *RBE* values for DSB induction under moderate hypoxia (2% O_2_) and severe hypoxia (0.1% O_2_) increase to the range of 1.3–2.9 and 1.4–4.4, respectively, indicating the advantage of GT for hypoxic tumor cells.

## Figures and Tables

**Table 1:** Absolute yields of DSBs (per Gy per Gbp) induced by ultrasoft X-rays and ^60^Co γ-rays.

Absolute Yield (per Gy per Gbp)	γ-ray Energy	Measured DSBs (per Gbp per Gy) [3]	*RBE*	MCDS DSBs (per Gbp per Gy)	*RBE*
Titanium K-shell	4.55 keV	10.4	1.4	10.7	1.3
Aluminum K-shell	1.49 keV	14.3	1.9	15.9	1.9
Copper L-shell	0.96 keV	17.4	2.3	18.5	2.3
Carbon K-shell	0.28 keV	20.7	2.7	21.2	2.6
^60^Co	1.17 MeV 1.33 MeV	7.6	1.0	8.1	1.0

DSBs, double-strand breaks; MCDS, Monte Carlo damage simulation; *RBE*, relative biological effectiveness.

**Table 2:** Absolute yields of DNA damage (per Gy per Gbp) induced by ultrasoft X-rays and ^60^Co γ-rays at a normal oxygen concentration (21%).

Absolute Yield (per Gy per Gbp)	γ-ray Energy	BD	SSB	SSB^+^	2SSB	DSB	DSB^+^	DSB^++^	Total SSBs	Total DSBs	Total Damage
Titanium K-shell	4.55 keV	365.0	160.7	13.3	3.1	7.7	2.2	0.8	177.1 ± 0.1	10.7 ± 0.0	552.8 ± 0.3
Aluminum K-shell	1.49 keV	257.8	129.8	18.7	6.1	9.4	4.1	2.3	154.6 ± 0.1	15.9 ± 0.0	428.3 ± 0.3
Copper L-shell	0.96 keV	207.8	114.8	19.9	7.2	10.1	5.1	3.2	141.9 ± 0.1	18.5 ± 0.0	368.2 ± 0.3
Carbon K-shell	0.28 keV	160.5	99.0	35.6	14.3	16.3	9.4	6.9	127.3 ± 0.0	21.2 ± 0.0	309.0 ± 0.1
^60^Co	1.17 MeV 1.33 MeV	424.4	178.6	8.0	1.0	7.1	1.0	0.1	187.4 ± 0.0	8.1 ± 0.0	620.2 ± 0.0

**Table 3:** Absolute yields of DNA damage (per Gy per Gbp) induced by ultrasoft X-rays and ^60^Co γ-rays under moderate hypoxia (2%).

Absolute Yield (per Gy per Gbp)	γ-ray Energy	BD	SSB	SSB^+^	2SSB	DSB	DSB^+^	DSB^++^	Total SSBs	Total DSBs	Total Damage
Titanium K-shell	4.55 keV	350.8	151.0	11.5	2.6	6.7	1.8	0.7	165.1 ± 0.0	9.1 ± 0.0	525.0 ± 0.2
Aluminum K-shell	1.49 keV	255.5	125.2	17.1	5.4	8.6	3.6	1.9	147.6 ± 0.1	14.1 ± 0.0	417.1 ± 0.3
Copper L-shell	0.96 keV	209.2	112.5	18.6	6.5	9.5	4.6	2.8	137.7 ± 0.1	16.8 ± 0.0	363.8 ± 0.3
Carbon K-shell	0.28 keV	163.2	98.5	34.5	13.5	16.0	9.0	6.4	125.7 ± 0.0	20.0 ± 0.0	308.9 ± 0.1
^60^Co	1.17 MeV 1.33 MeV	402.5	166.2	6.7	0.8	6.1	0.7	0.1	173.5 ± 0.0	6.8 ± 0.0	583.0 ± 0.0

**Table 4:** Absolute yields of DNA damage (per Gy per Gbp) induced by ultrasoft X-rays and ^60^Co γ-rays under severe hypoxia (0.1%).

Absolute Yield (per Gy per Gbp)	γ-ray Energy	BD	SSBS SSB	SSB^+^	2SSB	DSB	DSB^+^	DSB^++^	Total SSBs	Total DSBs	Total Damage
Titanium K-shell	4.55 keV	289.4	116.0	6.6	1.3	3.8	0.9	0.3	123.8 ± 0.0	4.9 ± 0.0	418.1 ± 0.1
Aluminum K-shell	1.49 keV	235.7	105.5	11.5	3.2	5.7	2.0	1.0	120.3 ± 0.0	8.7 ± 0.0	364.6 ± 0.2
Copper L-shell	0.96 keV	206.5	101.1	13.9	4.4	7.0	3.0	1.6	119.5 ± 0.0	11.5 ± 0.0	337.5 ± 0.2
Carbon K-shell	0.28 keV	170.9	95.0	30.0	10.8	14.5	7.3	4.5	117.4 ± 0.0	15.5 ± 0.0	303.8 ± 0.1
^60^Co	1.17 MeV 1.33 MeV	317.2	123.2	3.4	0.3	3.2	0.3	0.0	127.0 ± 0.0	3.5 ± 0.0	447.5 ± 0.0

**Table 5:** Average repair outcome probabilities for all types of DNA damage for cells irradiated with ultrasoft X-rays or ^60^Co γ-rays.

Radiation Type	Energy	Correct Repair	Mutation	DSB Conversion
Titanium K-shell	4.55 keV	0.934	0.049	0.017
Aluminum K-shell	1.49 keV	0.890	0.081	0.029
Copper L-shell	0.96 keV	0.863	0.099	0.037
Carbon K-shell	0.28 keV	0.829	0.123	0.048
^60^Co	1.17 MeV 1.33 MeV	0.963	0.0281	0.00907

**Table 6:** The relative biological effectiveness (*RBE*) of double-strand break (DSB) induction and enzymatic DSBs for long-patch base excision repair (LP BER) for cells irradiated with ultrasoft X-rays or ^60^Co γ-rays.

Radiation Type	γ-ray Energy	DSB Induction (per Gy per Gbp)	DSB Conversion (per Gy per Gbp)	*RBE* for DSB Induction	*RBE* for DSB Conversion
Titanium K-shell	4.55 keV	10.7	9.4	1.3	1.6
Aluminum K-shell	1.49 keV	15.9	12.6	1.9	2.1
Copper L-shell	0.96 keV	18.5	13.7	2.3	2.3
Carbon K-shell	0.28 keV	21.2	14.7	2.6	2.4
^60^Co	1.17 MeV 1.33 MeV	8.1	6.0	1.0	1.0

## Data Availability

Not applicable.
